# Environmental and Nutritional Parameters Modulating Genetic Expression for Virulence Factors of *Clostridioides difficile*

**DOI:** 10.3390/antibiotics13040365

**Published:** 2024-04-16

**Authors:** Zoe Masset, Sathursha Gunaratnam, Mathieu Millette, Lynne V. McFarland, Monique Lacroix

**Affiliations:** 1 INRS Armand-Frappier Health Biotechnology Research Centre, Research Laboratories in Sciences, 531 des Prairies Blvd, Laval, QC H7V 1B7, Canada; zoe.masset@inrs.ca (Z.M.); monique.lacroix@inrs.ca (M.L.); 2 Bio-K+, a Kerry Company, Preclinical Research Division, 495 Armand-Frappier Blvd, Laval, QC H7V 4B3, Canada; sathursha.gunaratnam@kerry.com (S.G.); mathieu.millette@kerry.com (M.M.); 3 Public Health Reserves Corps, Seattle, WA 98115, USA; 4 McFarland Consulting, Seattle, WA 98115, USA

**Keywords:** *Clostridioides difficile*, CDI, virulence, gene expression, environmental factors, antibiotics, probiotics

## Abstract

*Clostridioides difficile* infections (CDIs) continue to be a persistent healthcare concern despite newer antibiotic treatments, enhanced infection control practices, and preventive strategies focused on restoring the protective intestinal microbial barrier. Recent strides in gene sequencing research have identified many genes regulating diverse virulence factors for CDIs. These genes may be over- or under-expressed when triggered by various environmental and nutritional factors. The aims of this paper are to review the important genes involved in *C. difficile* pathogenesis and to identify modifiable environmental, nutritional, and other factors that may trigger the expression of these genes and thus offer new strategies to prevent CDIs.

## 1. Introduction

*Clostridioides difficile* infections (CDIs) have been a serious burden for healthcare systems for decades and continue to be a cause of outbreaks in hospitals. In the United States alone, CDI accounts for over 500,000 cases/year with 29,000 deaths [1,2]. Patients with CDIs typically have longer hospital stays (average 10 days) and higher intensive care unit admissions (up to 18%), resulting in higher healthcare costs and a heavier burden on healthcare workers [3]. Patients with an initial episode of CDI may also develop recurrent CDI (20–40%), which has a higher risk of developing sepsis and exposes patients to more antibiotic exposure for additional treatment [3].

Previous research initially focused on effective treatments for CDIs and, as more CDI outbreaks occurred, better strategies to control healthcare-associated outbreaks in hospitals [4]. During the last two decades, the roles of various toxins, risk factors, and the importance of the protective intestinal microbiome have been established [4]. The development of newer tools has expanded the scope of research into the field of genomic expression of virulence factors.

The pathogenesis of CDI involves a cascade of events: (1) disruption of the intestinal microbiome and metabolome, which acts as a protective barrier to the opportunistic invading *C. difficile* bacteria, (2) attachment of *C. difficile* to receptor sites on enterocyte cell surfaces, (3) reproduction of *C. difficile* reaching quorum sensing (QS) thresholds, which triggers the production of toxins, (4) toxin-mediated host cytoskeleton destruction, leading to enterocyte collapse, (5) opening of tight junctions, leading to fluid secretion, and (6) attraction of inflammatory cytokines to the site. A greater understanding of the pathogenesis cascade has led to strategies for the prevention and treatment of CDIs based on direct killing of the *C. difficile* bacterium with antibiotics or methods to restore the protective intestinal microbiome using probiotics or fecal microbial replacement. However, CDIs remain a global problem, and more innovative strategies to treat CDIs are urgently needed [4,5].

Recent research on the genome of the *C. difficile* chromosome has expanded our understanding of which genes are involved in the pathogenesis of CDI [6]. The emergence of a new “hypervirulent” strain (*C. difficile* NAP1/BI/027) was responsible for several outbreaks of CDIs in the 2000s [7]. The hypervirulent ribotype 027 strain has two distinguishing virulence determinants: the deletion of the *tcdC* gene, which is a negative regulator for toxin production, and a higher frequency of resistance to fluoroquinolones. The expansion of hypervirulent strains might have been due to genetic adaptations and environmental factors favoring the transmission of the virulence of *C. difficile*, but which environmental factors trigger genetic expression is still under investigation [6,8].

The aim of this paper is to review the most important genes involved in *C. difficile* pathogenesis, identify modifiable environmental, nutritional, and other factors that may trigger the expression of these genes, and thus offer new strategies to prevent CDIs.

## 2. Overview of Genes Associated with Virulence of *C. difficile*

A wide variety of genes have been found to be involved in the pathogenesis of *C. difficile*, including genes producing various toxins, regulator, and promoter genes, genes involved in QS cascades, and the production of important structural components of *C. difficile* cells (cell walls and flagella) and biofilm formation, as described in Table 1.

### 2.1. Production of Toxin A and Toxin B: tcdA and tcdB

**Figure 1:**
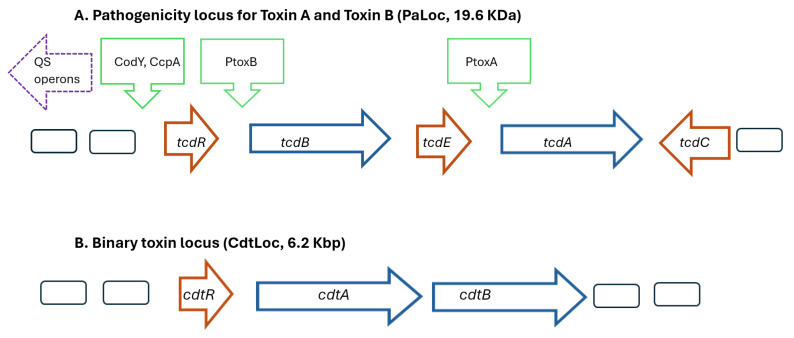
Schematic models showing the major genes present in the pathogenicity locus regions for (**A**) Toxins A and B (PaLoc) and (**B**) binary toxin (CdtLoc) of *Clostridioides difficile*. Promoter proteins indicated by green arrows, regulatory genes indicated by red arrows, and toxin genes indicated by blue arrows. The agr1 operon located upstream of the PaLoc operon involves genes regulating quorum sensing. Abbreviations: PtoxA Promoter toxin A; PtoxB Promoter toxin B; QS, quorum sensing.

The symptoms of CDIs result from the production of two exotoxins, which disrupt the cytoskeleton of the intestinal epithelial cells, and higher levels of toxins result in more severe CDIs [9,10]. Toxins A and B are encoded by the genes *tcdA* (308 kDa) and *tcdB* (270 kDa), respectively, and are located on the 19.6 Kb pathogenicity loci (PaLoc) on *C. difficile*’s chromosome (Figure 1A) [11]. The PaLoc possesses a mobile genetic element and is located at the same site in all toxigenic *C. difficile* strains [12]. Remarkable sequence diversity in genes that encode toxin B exists among different *C. difficile* strains and may contribute to differences in virulence [13]. Although non-pathogenic strains of *C. difficile* may lack of *tcdA* and *tcdB* genes, the PaLoc locus can be horizontally transferred, converting them into a pathogenic strains [14]. The toxin genes are transcribed when the accumulation of growth-inhibiting substances or nutrient limitation occurs, and QS systems upregulate toxin genes [9,15,16].

### 2.2. Regulatory Genes for Toxin A and Toxin B: tcdR, tcdC and tcdE

There are three important regulatory genes involved in the production of Toxin A and Toxin B. The first positive regulator gene (*tcdR)* codes for an RNA polymerase, TcdR (555 bp), which assists binding of promoters to the genes for Toxin A and Toxin B production [14]. The second positive regulator gene (*tcdE*) codes for a holin-like protein involved in the cell lysis and subsequent release of both Toxin A and Toxin B [6]. A negative regulator gene (*tcdC*) codes for an anti-sigma factor (TcdC) that downregulates *tcdR* expression and suppresses the production of Toxins A and B [17]. Increased virulence observed in the hypervirulent strains has been linked to mutations in the *tcdC* gene. A deletion of 18 bp in the *tcdC* gene enhances toxin production, resulting in more severe outcomes and higher mortality rates, highlighting the possible function of TcdC in regulating toxin expression in *C. difficile* [18].

### 2.3. Promoter Genes for Toxin A and Toxin B Production: ccpA, codY

Two promoter genes are involved in the production of Toxin A and Toxin B and are responsive to environmental factors. The gene *ccpA* produces a protein (CcpA), which binds to regulatory regions and reduces the production of these two toxins when sugar levels are low. CcpA is also involved in a diverse number of regulators involved in fermentation processes, especially for butyrate, which is known to increase Toxin A and B production. In addition, CcpA is involved in the sporulation process for *C. difficile* by repressing SpoOA and SigF, factors involved in the early development of spores. CcpA is also involved in the production of CodY.

The second promoter gene (*codY*) produces CodY, which is also involved in the expression of SpoOA. Like CcpA, CodY is involved in a wide range of transcription processes involving the production of Toxin A, Toxin B and the production of spores. CodY production is dependent upon nutrient levels in the environment [19,20,21].

### 2.4. Quorum Sensing Genes: agr1, agr2, luxS

Quorum sensing (QS) involves regulating gene expression in response to bacterial population densities. Different genes are involved in the *C. difficile* QS system depending upon whether the regulation occurs at an intraspecies level (*agr1* and *agr2*) or at an interspecies level (*luxS*) [15,22]. Once a threshold of bacterial density is reached, these genes produce autoinducers, which, in turn, stimulate the production of Toxin A and Toxin B [23,24]. The Agr1 locus is found in all virulent *C. difficile* strains. Hypervirulent strains (such as NAP1/027 R20291) contain an additional Agr2 locus [15,25,26]. The *luxS* gene produces LuxS, an enzyme that produces auto-inducers that upregulate the expression of *tcdA*, *tcdB,* and *tcdE*, resulting in an increase in Toxin A and B production.

### 2.5. Binary Toxin Genes: cdtA and cdtB

A third type of toxin is produced by some strains of *C. difficile* called binary toxin (CDT), made of two polypeptides: CDTa (48 KDa) and CDTb (75 KDa). The *cdtA* gene encodes CDTa, a G-actin-specific ADP-ribosyl transferase that disrupts the actin cytoskeleton, leading to cell death. The *cdtB* gene encodes CDTb, which acts to internalize CDTa into the interior of the host cell [27]. The genes for these polypeptides are found on a separate locus (CdtLoc) distinct from the PaLoc locus (Figure 1B). A regulatory gene (*cdtR*, 747 bp) encodes a 30 KDa protein (CdtR) which results in higher levels of binary toxin [27]. Some strains producing binary toxin but not having the CdtLoc operon have a unique 68 bp sequence at the location but the function has not been well defined [28].

### 2.6. Adhesion Genes: cwp13, cwp66, cwp84, Etc

The *C. difficile* S-layer is composed of numerous proteins, and the surface layer protein A (SlpA) is essential for the attachment of *C. difficile* to intestinal epithelial cells. The different Slps, including SlpA, are part of a class of cell wall proteins (CWPs), including Cwp66, Cwp84, Cwp13, and CwpV, proteins that are involved in the assembly of the S-layer [29,30,31].

### 2.7. Biofilm Genes: spoOA

*C. difficile* colonizes the human gut and can persist through its ability to form biofilms, a complex process involving many factors [32,33,34]. Bacteria within biofilms can be more resistant to antibiotics than their planktonic counterparts via several mechanisms. However, the opposite has also been observed since antibiotic molecules can be more concentrated in biofilms [35]. An increased resistance has been observed for *C. difficile* strains within biofilms [36]. The protein Spo0A is an important contributor to the development of biofilms in *C. difficile*. It is also a critical factor in persistent infections, including CDI recurrences [36]. High concentrations of Spo0A are involved in sporulation, while intermediate levels activate genes shaping the biofilm matrix [37]. The presence of another protein, LuxS, has been linked with biofilm formation [38].

### 2.8. Motility Genes: fliC, fliE, filG

Some strains of *C. difficile* have flagella. These flagella allow movement from one site to another in the gut. The appendages are considered virulence factors and are involved in the modulation of toxin production [39]. FliC, a 39-kDa flagellin protein, and FliD, a 56-kDa flagellar cap protein, are critical structures for *C. difficile* adherence in gut cells. FliC and FliD have also been shown to trigger an inflammatory response [40,41].

### 2.9. Genes Involved in Spore Germination: cspA, cspC, spoOA

The formation of spores allows *C. difficile* to resist contact with antibiotics, oxygen, heat, and common disinfectants. The germination of *C. difficile* spores depends on the upregulation or downregulation of more than 500 genes on the cspBAC gene locus. Primary bile acids, such as taurocholate, bind to Cspc receptor sites on spore surfaces, followed by calcium and dipocolinic acid release and phosphorylation and activation of the transcription factor Spo0A, which directly regulates dozens of genes [42].

## 3. Modifiable Parameters of *C. difficile* Gene Expression

### 3.1. Antibiotics

Antibiotics are the most common risk factor for antibiotic-associated diarrhea and CDIs [43]. Historically, the elimination or reduction in protective bacterial species in the intestinal microbiome barrier by antibiotics was the accepted mechanism of this risk factor [17,24,43]. However, as our appreciation of the roles different genetic expressions have on *C. difficile* virulence has grown, the possibility that antibiotics may impact the host by other mechanisms is being explored.

#### 3.1.1. Antibiotics and Toxin A and Toxin B Production

Recent studies have demonstrated the impact of various environmental factors on the expression of *tcdA* and *tcdB* genes in *C. difficile* [9]. Additionally, antibiotics have been shown to alter the pattern of toxin gene expression in *C. difficile*, as evidenced by in vitro experiments utilizing sub-minimal inhibitory concentrations (MIC) of different antibiotics. Metronidazole, vancomycin, clindamycin, and linezolid have been observed to enhance the transcription rate of genes for Toxin A and B during the exponential phase of bacterial growth in all tested antibiotics, except clindamycin, with *C. difficile* VPI 10463 (ATCC 43255), strain 2932 (which has a deletion in the PaLoc thus not able to produce Toxin A), strain 47, and strain 317 [44]. It has been demonstrated that the transcription of toxin genes varies depending on the *C. difficile* strain and the type of antibiotics [45].

*C. difficile* strain 039 is resistant to ciprofloxacin, and dose-dependent subinhibitory concentrations of ciprofloxacin have been found to significantly increase Toxin A gene expression and shift its expression to earlier in its growth cycle [46]. While a high concentration of ciprofloxacin increased *tcdB* gene expression, it displayed less sensitivity to low-dose ciprofloxacin. In clinical isolates of *C. difficile* strains 5325 and BI/NAP1/027, ciprofloxacin at 0.25 MIC markedly boosted both *tcdA* and *tcdB* gene expression, albeit their temporal dynamics are similar as compared to the control [46].

Tigecycline, a protein synthesis inhibitor, was studied in vitro in cultures of *C. difficile* strain 9689 and hypervirulent BI/NAP1/027 (strain 5325) isolates to verify the impact on Toxin A and B production. The antibiotic induced more expression of Toxin A and B genes, and this expression was observed faster during the culture of both strains [47].

During the stationary phase of *C. difficile* strains UK-1 and CD196, fidaxomicin inhibited the transcripts from the pathogenicity loci (*tcdR*, *tcdA*, and *tcdB*), while vancomycin did not [48].

#### 3.1.2. Antibiotics and Adherence

The initial phase of infection hinges on the adherence to epithelial cells. In a comprehensive study, both toxigenic and non-toxigenic *C. difficile* isolates were exposed to a range of antibiotics [49]. Notably, exposure to clindamycin and ampicillin prompted a notable increase in the expression of genes encoding colonization factors across all six strains. However, the extent of gene regulation exhibited significant variability among the strains, with *cwp84* having the most pronounced upregulation following antibiotic exposure. Conversely, the presence of ofloxacin, moxifloxacin, or kanamycin resulted in minimal alterations in the expression of these genes [49].

In another investigation, the impact of four antibiotics on the expression of genes encoding three colonization factors was examined in four NAP1/027 strains, including one moxifloxacin-susceptible and three moxifloxacin-resistant strains [50]. Interestingly, exposure to ampicillin or clindamycin substantially heightened the expression of *cwp84* and *slpA* in NAP1/027 strains. However, following exposure to fluoroquinolones, increased expression of *cwp84* and *slpA* was only observed in moxifloxacin-resistant strains. These findings underscore the potential of fluoroquinolones to selectively promote the expression of specific colonization factor-encoding genes in resistant *C. difficile* strains.

#### 3.1.3. Antibiotics and Biofilm Formation

Fidaxomicin and its major metabolite OP-1118 can also be used to treat CDIs and has lower recurrence rates compared with vancomycin [51,52]. Fidaxomicin inhibited biofilm formation by influencing *fliC* transcription of *C. difficile* UK027, unlike vancomycin, which did not influence *fliC* mRNA expression [52].

#### 3.1.4. Antibiotics and Spore Germination

Tigecycline induced a notable increase in *spo0A* transcription in *C. difficile* 9689. However, despite this transcriptional upregulation, viable spore formation was consistently reduced across all concentrations of tigecycline. Conversely, in the hypervirulent NAP1 5325 strain, tigecycline did not significantly affect the already elevated level of *spo0A* transcription at 48 h. Nevertheless, it significantly suppressed spore formation in this strain [47].

### 3.2. Nutritional Factors

The metabolic network and the nutritional status in the environment play important roles in the growth and expression of pathogenicity *of C. difficile* [53]. The production of toxins is dependent on the nutrient levels in the growth medium and environmental signals, such as low levels of phosphotransferase system sugars, biotin, and amino acids, especially cysteine [42,54]. The effects of nutritional limitation are varied; indeed, in a complex media, the presence of rapidly metabolizable carbon sources results in lower toxin production, while in contrast, the limitation in biotin leads to an increase in Toxin A and B production [55].

#### 3.2.1. Amino Acids

There are several amino acids capable of regulating the production of *C. difficile* toxins. However, cysteine remains the most effective [56]. Cysteine negatively regulates several metabolic pathways, including the production of butyric acids and butanol, leading to a reduction in toxin production [54,56]. Cysteine affects many genes linked to NAP1/BI/027, as well as flagellar and ribosomal genes [56]. Moreover, in response to cysteine, the synthesis of toxins and the production of butyrate may be controlled in different ways [56].

#### 3.2.2. Carbohydrates

Thanks to the activity of nutrient-sensitive regulators such as CcpA and CodY, certain sugars and peptides can suppress the expression of *C. difficile* toxin [9]. The CcpA regulon includes genes involved in fermentation, amino acid metabolism, and sugar absorption, confirming its role in linking carbon and nitrogen pathways [57,58,59]. The inhibition by CodY and CcpA of toxin and nutrient acquisition mechanisms is disrupted when these peptides and simple sugars are depleted. Glucose regulates approximately 20% of all *C. difficile* genes, with 50% tributary to CcpA for their regulation [57].

#### 3.2.3. Green Tea

Epigallocatechin gallate, present in green tea, was found to downregulate the production of LuxS involved in QS cascades and cause a decrease in the amount of AI-2 and a lower expression of the *tcdA* and *tcdR* genes [60].

#### 3.2.4. Bile Acids

The primary bile acid taurocholate can act as a co-germinant of the spores with the glycine, and another primary bile acid (deoxycholate) also supports germination and suppresses vegetative growth. In contrast, chenodeoxycholate inhibits spore germination by the action of taurocholate and suppresses vegetative growth at the same time [61]. The commensal microbiota plays a vital role in the production of secondary bile acids. Most probiotics have been shown to metabolize primary bile acids into secondary bile acids, which do not stimulate spore germination. In a randomized control trial, administration of secondary bile acid to 16 patients with recurrent CDIs resulted in a cure rate of 88% [62].

### 3.3. Environmental Stimuli

Bacteria must adapt to changes in temperature, nutrient deprivation, radiation, acidity, water scarcity, and more [63]. Variations in gene expression form the primary aspect of bacterial responses to stress and alterations in the environment, engaging diverse mechanisms [64]. Bacteria must constantly adapt physiologically via drastic changes in gene expression. Indeed, environmental conditions are constantly evolving.

#### 3.3.1. Stress

Environmental stresses such as oxidative, nutritional, and osmotic stress can cause damage to *C. difficile* DNA compromising its genetic stability and ability to survive in adverse conditions. Proteins play a role in DNA repair when responding to stress. Mfd, a highly conserved bacterial protein, connects DNA repair with transcription. Mutations in the *mfd* gene of *C. difficile* result in a notable rise in the production of Toxins A and B and abnormal colony morphology. Enhanced transcription of the *tcdA* and *tcdB* genes is noted, implying the relaxation of transcriptional repression mediated by CcpA in the *mfd* mutant CodY. It is possible that Mfd facilitates the release of RNA polymerase molecules obstructed by barriers established by CodY and CcpA [65].

#### 3.3.2. Temperature

Changes in temperature can affect gene expression in bacteria; these changes lead to upregulation of the expression of virulence determinants in many pathogens [66]. Increasing the temperature from 22 to 37 °C showed positive upregulation in *tcdA* and *tcdB* with an increase in toxins in the medium in certain strains of *C. difficile* [67]. These observations show the impact of temperature at the transcriptional level. Toxin production in *C. difficile* is temperature-dependent and is optimal at 37 °C. An alternative sigma factor, TcdD, encoded by a gene located in PaLoc immediately upstream of *tcdB*, is required for the temperature-dependent activity of the toxin promoters. Temperature was also found to affect twelve proteins that were previously regulated during biotin deficiency. They matched the criteria for having the highest expression at 37 °C and, thus, high toxin production. Upregulation of toxin synthesis with limitation of biotin and glucose-amino acids occurred at 37 °C but not at 22 °C or 42 °C, indicating that temperature regulation can operate at a basal level [67]. Biotin limitation also causes slower growth and high toxin production, but this can be reversed by adding amino acids such as asparagine, glutamine, or lysine in the medium. Levels of butyric acid production are also regulated by temperature. As for the toxins, expression of 3-hydroxybutyryl-CoA dehydrogenase, a key enzyme in butyric acid production, was at the highest at 37 °C, and the biggest difference (>10-fold) was found between the 22 °C and 37 °C. Other short-chain fatty acids like acetic or propionic did not demonstrate a temperature dependency.

#### 3.3.3. pH

Low pH levels have been implicated in the upregulation of tcdA in some strains of *C. difficile*, like NAP1/BI/027, but not all *C. difficile* strains were sensitive to low pH levels in vitro [68]. Lower pH levels have also been found to result in lower sporulation rates [6]. Protein pump inhibitors are considered a risk factor for CDIs, thus highlighting the importance of pH levels, but the role has not been definitively proven [43,69].

### 3.4. Viral Factors: Prophages

Bacteriophages play a role in the development of most bacteria. Lysogenic phages have the ability to neutralize their host via the reproduction cycle and can create a stable parasitic connection with their host via a lysogenic cycle [70]. *C. difficile* may carry more than one prophage in most strains, some of which, such as ϕCD38-2 and ϕCD119, have the ability to modulate the expression of virulence genes [71]. The cpC6 phage can decrease the transcription of *tcdA* [72]. In addition, the lysogenization of *C. difficile* strains by the ϕCD119 phage results in a decrease in the production of Toxins A and B and the transcription of the PaLoc (*tcdA, tcdB, tcdE, tcdR* and *tcdC*). Indeed, a phage repressor, encoded by the *repR* gene, binds to the promoter region upstream of the *tcdR* gene, a positive transcriptional regulator gene of the pathogenicity locus. This interaction results in repressing the transcription of PaLoc genes and affects the production of Toxins A and B. Thus, phages are able to influence major bacterial phenotypes, such as toxin production [71].

Recently, a complete binary toxin locus (CdtLoc) was discovered in the genome of the phiSemix9P1 phage. This phage has been shown to carry a complete binary toxin locus. Additionally, several regulatory genes have been identified in the genomes of these phages, suggesting that their interactions with the host may be complex and often nuanced [73]. Phages can also be involved in cell-to-cell communication via QS. Research has shown that three homologs of bacterial genes involved in QS (*agrB*, *agrC*, and *agrD*) are encoded by the PhiCDHM1 prophage [74]. This implies that *C. difficile* prophages could express genes capable of influencing bacterial communities. The study of gene expression in biofilms of wild-type strains and *luxS* mutants indicated a negative regulation of prophage loci in *luxS* mutant biofilms [31]. The release of DNA by phage-mediated cell lysis likely contributes to the formation of *C. difficile* biofilms [38].

### 3.5. Microbial Factors: Probiotics

The treatment of patients with antibiotics is a standard treatment for CDIs, but probiotics have also been used to treat and prevent CDIs [75,76]. The most utilized types of probiotics are usually in the family *Lactobacillaceae* or within the genera *Bifidobacterium* spp. or yeast (*Saccharomyces* spp.), which show different efficiencies with CDIs. The efficacy and mechanisms of action of probiotics in regulating intestinal microbiota functions are both disease-specific and strain-specific [77]. All probiotic strains do not fight against all pathogens, as not all probiotic strains possess the same type of mechanisms, and one probiotic strain may be effective for different pathogens, depending on the type of pathogen-specific defenses it possesses. The mechanisms can be diverse, including the production of antimicrobial metabolites, bacteriocins, organic acids or short chain fatty acids (SCFAs) as well as restricting pathogenic growth by competing for the nutrients and adherence on the mucosal barrier in the gut, production of toxin-destructive proteases and the regulation of the immune response [69]. Lactic acid bacteria (LAB) such as lactobacilli are beneficial microorganisms present in healthy human microbiota and have been associated with immunomodulatory effects, reduction in pathogenic bacteria levels, reduction in antibiotic-associated gastrointestinal symptoms, reduction in acute diarrhea, inflammatory bowel disease, and allergy symptoms [78,79]. These mechanisms have been well-studied for a variety of probiotic types, but whether probiotics can affect *C. difficile* on a genetic level has not been well documented.

Technological advances allow us to study all messenger RNAs produced during the transcription process of the *C. difficile* genome, notably in contact with probiotic strains. The study of gene expression of *C. difficile* is essential to understanding the mechanisms of action of probiotics against *C. difficile*. At transcriptional levels for virulence-associated genes in *C. difficile* on ribotypes 027, 078, and 001, the *luxS* and *txeR* genes are decreased with the presence of *Lactobacillus acidophilus* GP1B [80]. These reduced levels of expression appeared to affect the transcription of *tcdA* and *tcdB* genes. Moreover, the downregulation of luxS induces the reduction in AI-2 levels. The addition of a cell extract of *L. acidophilus* GP1B resulted in the downregulation of virulence genes in *C. difficile* at the level of mRNA. *L. acidophilus* GP1B is not the only probiotic that can impact *C. difficile* gene expression.

*L. acidophilus* La-5 secretes bioactive molecules namely proteobiotics, which also causes a significant downregulation of *tcdA* and *tcdB* and *cwp84* in *C. difficile* ribotypes 027, 078, and 001 [81].

*Lactobacillus fermentum* Lim2 isolated from kimchi can affect the QS and virulence factors of *C. difficile* 027. A gene expression analysis indicated that the presence of 100 mg per mL or heat-inactivated cell extract significantly suppressed the QS (*luxS*) and the virulence factors (*tcdA, tcdB,* and *tcdE*) while upregulating the negative regulator gene (*tcdC*) [82].

A three-strain probiotic blend (*L. acidophilus* CL1285, *Lacticaseibacillus casei* LBC80R, *Lacticaseibacillus rhamnosus* CLR2, Bio-K+) was found to alter genetic expression in *C. difficile*. A study detected 1156 differently expressed genes in *C. difficile* with this Lactobacilli mixture. The effect of the Lactobacilli probiotic included the overexpression of CDT binary toxin genes, underexpression of *fliC* gene and other flagella genes, up- and downregulation of different sporulation genes (*spoOA*), reduction in QS (*luxS*) genes, up- and downregulation of different *cwp* adhesion genes, but no effect on Toxin A or B gene expression [83].

Other genus of bacteria, such as *Bifidobacterium* spp., can modulate the expression of *C. difficile* genes. *Bifidobacterium breve* YH68 finds extensive application in food fermentation and biomedical fields. The cell-free supernatant (CFS) of *B. breve* YH68 notably decreased the gene expression levels of *tcdA* and *tcdB* in *C. difficile* ATCC 9689 [84]. Previous research has linked the expression of *tcdA*/*B* to the production of autoinducer-2 (AI-2) by *C. difficile*, an important structure of its quorum sensing (QS) system [80]. Consequently, *B. breve* YH68 CFS curbed *C. difficile* pathogenicity by inhibiting QS, subsequently leading to the downregulation of both *tcdA* and *tcdB* [84,85]. A more recent transcriptomic investigation explored the impact of varying doses of *B. breve* YH68 (10^8–^10^12^) on *C. difficile* ATCC 9689 [86]. Both doses of *B. breve* YH68-CFS induced significant alterations in activities in these metabolic pathways [86]. Notably, the high dose of probiotics led to significant suppression of genes associated with QS and signal transduction while enhancing those encoding toxin production and sporulation factors. Conversely, the low dose of *B. breve* resulted in the suppression of genes related to flagellar assembly (e.g., *fliD*, *fliC*, and *flgE*) and biofilm formation, alongside significant upregulation of drug resistance-related genes.

Investigations of other types of Bifidobacterial and Lactobacilli probiotics on *C. difficile* gene expression involved in QS cascade pathways are also ongoing [78].

## 4. Discussion

Our review found several types of environmental and nutritional factors that act as triggers for upregulating or downregulating the genetic expression of genes involved in the virulence pathways of *C. difficile*. This is an important finding to clinicians and researchers, as these triggers may be amenable to modifying the risk of patients from developing CDIs and may provide new therapeutic strategies for the treatment of CDIs. However, it is apparent that further research is needed to understand how and when various genes are expressed and if there are additional triggers that can be identified. Future studies could also investigate if these diverse factors act synergistically or if there are significant interactions between them. The strengths of our review include an extensive search of the literature relating to the genetic expression of *C. difficile* virulence factors and associated factors that might trigger the transcription of these genes. In addition, this review had the original goal to link these triggers with the known roles of the genes involved in the pathogenesis of *C. difficile*. Limitations of this review include the relative scarcity of studies linking environmental triggers to gene expression. Also, we did not include small non-coding RNA regulators in our review (such as Hfq and KhpB), as we could not find a link with any environmental or nutritional factors [87,88]. Continued research into these fields would be most useful.

## 5. Conclusions

Recent investigations into the genetic expression for *C. difficile* virulence factors have revealed a deeper understanding of the pathogenesis of this infection. In addition to unraveling how and when *C. difficile* toxins are produced, our understanding of the importance of other types of genes involved in pathogenesis, such as genes for adhesion, biofilm formation, motility, and spore germination, has increased. A wider appreciation for the diversity of triggers for gene expression, including antibiotic exposure, nutritional factors, environmental factors, prophage, and probiotic strains, has been gained by recent studies. Translating these results into clinical strategies for CDIs will require future studies but offers enticing alternatives to current therapies.

## Figures and Tables

**Table 1:** Genes involved in the pathogenesis of *C. difficile* and factors that influence their expression.

Genes	Codes for	Roles	Factors Impacting Genetic Expression
*tcdA* *tcdB*	Toxin A (308 KDa) Toxin B (270 KDa)	enterotoxin and cytotoxins, inactivates Rho GTPases, resulting in actin filament disorganization, loss of cytoskeleton results in enterocyte collapse, opening tight junctions, with fluid secretion and diarrhea; also promotes inflammatory response	antibiotics, amino acids (cysteine), carbohydrates, green tea components, stress, temperature, prophage, probiotics
*tcdR*	RNA polymerase sigma factor (555 bp)	positive regulator for Toxins A and B	
*tcdE*	holin-like protein (501 bp)	positive regulator that facilitates extracellular release of toxins A and B via lytic action	
*tcdC*	anti-sigma factor (699 bp)	negative regulator for Toxin A and B production by disruption of TcdR	
*codY*	promoter for transcription (1113 bp)	binds to *tcdR*	nutritional (butyrate)
*ccpA*	promoter for transcription (831 bp)	binds to *tcdR*	nutritional (glucose)
*agr1, agr2*	intraspecies accessory gene regulators (1500 bp, 500 bp)	QS pathway	nutritional (green tea), probiotics
*luxS*	interspecies gene regulatory (552 bp)	QS pathway	probiotics
*cdtA* *cdtB*	binary toxins (CDT), CdtA is an ADP-ribosyltransferase (1785 bp), CdtB is a polypeptide (1956 bp)	CdtA depolymerizes actin cytoskeleton, CdtB binds to host cells and transports CdtA into host cells	prophages, probiotics
*cdtR*	response regulator for CDT (747 bp)	positive regulator *cdtA*/*cdtB*	
*cwp13, cwp66, cwp84, cwpV, etc.*	cell wall proteins (2982 bp, 3168 bp, 2040 bp, etc.)	adhesion on S-layer of *C. difficile* cell wall	antibiotics, stress, probiotics
*slpA*	major S-layer constituent (885 bp)	cellular structure	antibiotics, stress, probiotics
*spoOA*	protein (1500 bp)	biofilm formation	antibiotics, carbohydrates
*fliC, fliE, fliG*	flagellar proteins (1155 bp, 1053 bp, 1317 bp)	motility and toxin production	antibiotics, probiotics
*cspA, cspC, spoOA*	regulatory genes (216 bp, 168 bp, 1509 bp)	spore germination, bile acid receptor	antibiotics, bile acids, environmental pH, probiotics
*sigL*	sigma factor regulator (426 bp)	downregulates Toxin A and B production	nutrition (cysteine)

Units: bp, basepairs; KDa, kilodaltons.
